# Histopathological Gap in Aortic Diseases: A Prospective Analysis

**DOI:** 10.3390/ijms242015470

**Published:** 2023-10-23

**Authors:** Cosmin Marian Banceu, Simona Gurzu, Catalin-Bogdan Satala, Dana Ghiga, Mihai Halic Neamtu, Vladimir Voth, Markus Liebrich, Horatiu Suciu

**Affiliations:** 1 I.O.S.U.D., George Emil Palade University of Medicine, Pharmacy, Science, and Technology of Targu Mures, 540139 Targu Mures, Romania; cosmin.banceu@umfst.ro; 2 Department of Surgery M3, George Emil Palade University of Medicine, Pharmacy, Science, and Technology of Targu Mures, 540139 Targu Mures, Romania; horatiu.suciu@umfst.ro; 3 Emergency Institute for Cardiovascular Diseases and Transplantation Targu Mures, 540136 Targu Mures, Romania; 4 Department of Pathology, George Emil Palade University of Medicine, Pharmacy, Science, and Technology of Targu Mures, 540139 Targu Mures, Romania; catalin.satala@umfst.ro; 5 Research Center for Oncopathology and Translational Medicine (CCOMT), George Emil Palade University of Medicine, Pharmacy, Science, and Technology of Targu Mures, 540142 Targu Mures, Romania; 6 Romanian Academy of Medical Sciences, 030167 Bucharest, Romania; 7 Department of Medical Scientific Research Methodology, George Emil Palade University of Medicine, Pharmacy, Science, and Technology of Targu Mures, 540139 Targu Mures, Romania; dana.ghiga@umfst.ro; 8 Swiss Federal Institute of Forest, Snow and Landscape Research WSL, 8903 Birmensdorf, Switzerland; nemarius@ethz.ch; 9 Institute of Environmental Engineering, ETH Zurich, 8039 Zurich, Switzerland; 10 Sana Cardiac Surgery, Herdweg 2, 70174 Stuttgart, Germany; vladimir.voth@sana.de (V.V.); markus.liebrich@sana.de (M.L.)

**Keywords:** aortic dissection, aortic aneurysm, histology, surgical replacement, myxomatous

## Abstract

Aortic dissection (AD) is a critical cardiovascular condition with the potential for devastating consequences. This study evaluated the histological changes in the aorta wall in patients with AD and aortic aneurysm (AA) who received surgical aortic replacement. Histopathological data showed that modifications of the media layer (*p* = 0.0197), myxomatous aspect (*p* = 0.0001), and subendothelial layer degeneration (*p* = 0.0107) were more frequently seen in AA versus AD samples. Patients with AA were approximately twice as likely to develop histological changes than those with AD (*p* = 0.0037). Patients with moderate or severe medial degeneration had a higher chance of developing AD (*p* = 0.0001). Because the histopathological score proved to be a predictor of both in-hospital and overall mortality, its evaluation should become the standard of care in any patients who undergo aortic replacement. Individualized postoperative management might be influenced by the histopathological aspect of the aortic layer.

## 1. Introduction

Acute aortic dissection (AD) is a life-threatening disease in which the risk of mortality increases by 1% per hour without surgical replacement. The stepwise pathomechanism usually includes rupture of the intima with subsequent rupture of the media and adventitia of the aortic layer [1,2,3]. AD might be anterograde or retrograde and longitudinal or transverse. Longitudinal dissection usually causes several re-entries and exit tears. Transverse dissection conducts the progression of the dissection process at the adventitia until its rupture with extravasation of blood in the aortic lumen. Consequently, transverse dissection is associated with a higher risk of preoperative death [4,5,6,7,8].

How the type of dissection or the histopathological changes might influence the mortality rate is unclear. Although several histological studies have focused on AD, the histogenesis and the predictive value of histological assessment are far from understood. Cystic medial necrosis, described by Erdheim [9], was long considered the starting point of dissection. Other associated events include the degeneration of the vasa vasorum, which has been hypothesized to induce hypoxia and increase the risk of aortic rupture [10]. Osada et al. suggested that the absence of restorative fibrosis with significant collagen concentration and perifocal medial apoptosis is another risk factor for aortic rupture [11].

In this context, the Society for Cardiovascular Pathology and the Association for European Cardiovascular Pathology, based on studies by Halushka et al., elaborated the principles that can be used for the histological evaluation of medial degeneration. However, this classification requires time to prove its effectiveness and be unanimously accepted by the community [12]. In this framework, the medial degeneration and the lesions of the aortic media layer can be evaluated using hematoxylin–eosin (HE) and other histochemical stains such as trichrome, van Gieson, van Gieson–elastica, and PAS Alcian blue. These might be helpful in assessing medial fibrosis, smooth muscle cell nuclei loss or cell disorganization, laminar medial collapse, elastic fiber thinning, disorganization, fragmentation, and mucoid extracellular matrix accumulation (MEMA) with its subdivisions: interlamellar (MEMA-i) and translamellar (MEMA-t). Based on the aforementioned parameters, medial degeneration can be classified as mild, moderate, or severe [12].

This study aimed to provide an in-depth analysis of the histological assessment of the aortic wall and the impact of histological changes on in-hospital mortality and overall mortality in AD patients who received an ascendant aorta replacement. They were compared to patients who received a replacement for an aortic aneurysm (AA).

## 2. Results

### 2.1. Clinicopathological Features and Study Design

**Figure 1:**
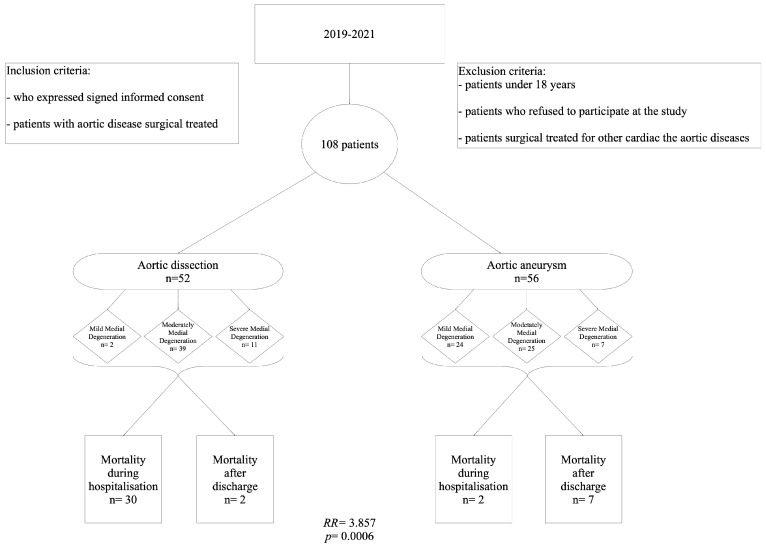
Stepwise approach used in the study.

Most of the 108 patients included in the study were men (Figure 1). The male:female ratio was 2.6:1 in both groups. Although most of the patients were aged over 55 years, those in the AA group were significantly younger. The hospitalization time was similar for the two groups (Table 1).

The rate of preoperative complications was significantly higher in the AD group but there were no differences regarding the postoperative complications (Table 2).

A significant association was found between AD and in-hospital mortality rate. Patients with AD had a 3.857 times higher risk of in-hospital mortality than those with AA (RR = 3.857, 95% CI: 1.127–13.196, *p* = 0.0006; Figure 1).

**Figure 2:**
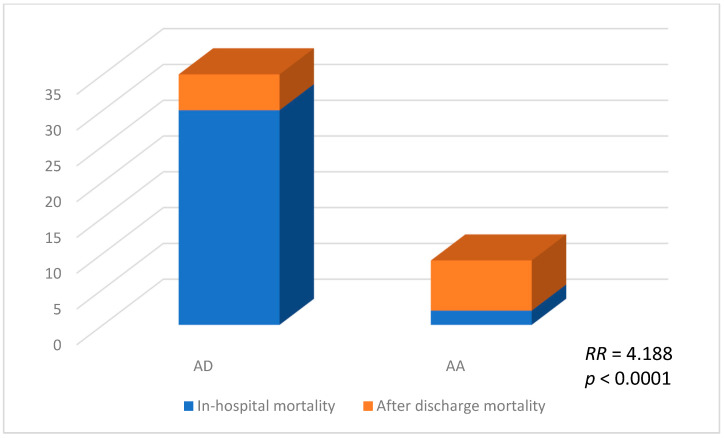
Representation of overall mortality.

We additionally analyzed whether AD was a risk factor for overall mortality, and a statistically significant association was observed. Patients with AD had a 4.188 times higher risk of death (RR = 4.188, 95% CI: 2.235–7.847, *p* < 0.0001; Figure 2).

### 2.2. Histopathological Changes: Description

**Figure 3:**
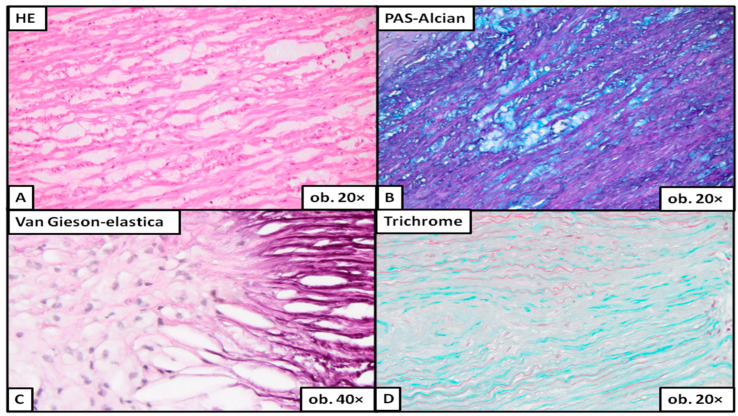
Aortic wall with severe medial degeneration. The multifocal MEMA breaks across the lamellar units (**A**), disrupting the continuity of elastic fibers and expanding into the adjacent units (**B**). The elastic lamellae are completely destroyed (**C**), and the architectural distortion is enhanced by fibrosis (**D**).

**Figure 4:**
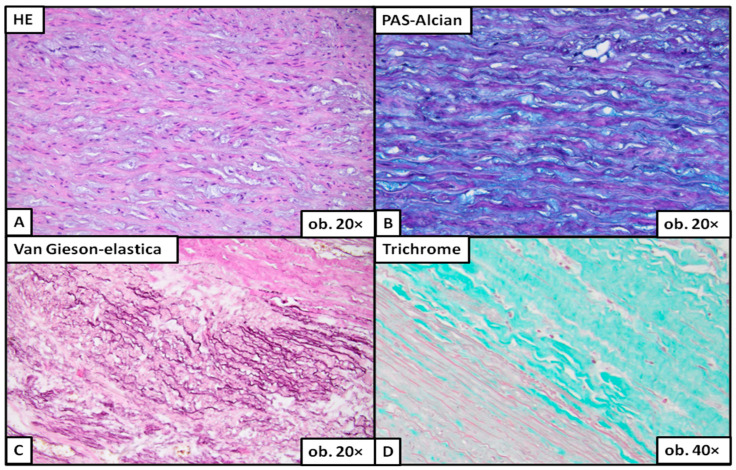
Aortic wall with moderate medial degeneration. The normal architecture of the aortic wall is effaced due to matrix accumulation (**A**,**B**), which leads to fragmentation of the elastic fibers in some areas where MEMA is more pronounced (**C**). The proliferation of collagen fibers is more evident than the fibrosis encountered in mild medial degeneration (**D**).

**Figure 5:**
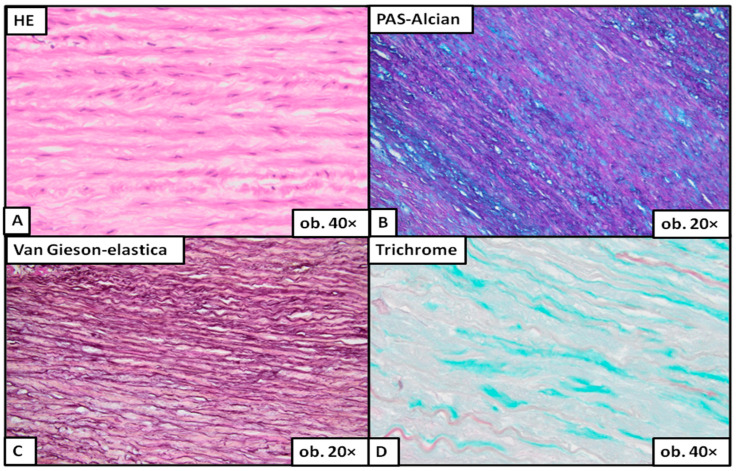
Aortic wall with mild medial degeneration: longitudinal sections. The mucoid accumulation causes the expansion of the intralamellar space, visible on HE (**A**) and PAS Alcian staining (**B**). MEMA compresses the elastic fibers surrounding the lamellar units, which appear thinner and collapsed (**C**). Focal fibrotic areas can be observed on the trichrome stain (**D**).

Under the microscope, severe degeneration (Figure 3) was easily detected on the classic HE stain. To evaluate moderate (Figure 4) or mild degeneration (Figure 5), the use of histochemical stains was necessary. PAS Alcian blue proved useful as the elective stain for mucoid medial change, either MEMA-i or the more severe form, MEMA-t. Medial fibrosis was emphasized with a trichrome stain, and elastic fiber fragmentation and loss were visualized with van Gieson–elastica and trichrome stains (Figure 3, Figure 4 and Figure 5).

### 2.3. Histopathological Changes: Quantification

MEMA-i was the most common histological degenerative change. A mild degenerative degree was observed only in the AA group (n = 6); no patient had a mild degree in the AD group. A moderate score was more prevalent in the AA samples than the AD samples (n = 24 vs. n = 9), whereas the opposite was true for the severe degenerative degree (n = 26 for the AA group vs. n = 43 for the AD group).

Regarding the MEMA-t parameter, a higher mild degree was observed for the AA group (n = 20) versus the AD group (n = 4). Moderate scores were observed in 79.62% of AD patients compared to 48.00% of AA patients, whereas severe scores were similar for the two groups.

Elastic fragmentation and loss changes were observed with mild scores in 11 patients of the AD group versus 29 of the AA group. More AD patients (n = 37) had a moderate degree than in the AA group (n = 20). Data analysis showed a statistically significant association between AD and the type of elastic fragmentation or loss (*p* = 0.0011).

A further histological degenerative change analyzed was smooth muscle cell nuclei loss. As shown in Table 3, AA patients had a higher mild degenerative degree (70.27%) compared to AD patients (50.98%), whereas the opposite was true for the mild degree (AD with 45% vs. AA with 27%). A statistically significant association was observed between AD and the severity of smooth muscle cell nuclei loss (*p* < 0.0001).

Finally, we focused on laminar medial collapse. The AD group exhibited higher mild scores (45.83%) compared to the AA group (60.00%), whereas the opposite was true for the moderate scores (45.83% vs. 35.00%). Regarding the severe scores, the AD group had a slightly higher probability compared to the AA group (8.33% vs. 5%).

The cases were further subdivided based on multifocal and focal changes. Mild multifocal laminar medial collapse occurred in 14 patients (73.68%) in the AD group versus five patients (26.32%) in the AA group. Mild focal changes occurred in eight patients (29.63%) in the AD versus 19 patients (70.37%) in the AA group. Using the risk ratio (RR) of 3.055 (95% CI: 1.317–7.087), patients with AD were over three times more likely to develop laminar medial collapse with mild multifocal changes than patients with AA (Table 4).

Only 7.69% (n = 2) of patients with mild medial degeneration developed AD, in contrast to 92.31% (n = 24) who had AA. The frequency of AD was higher among patients with moderate or severe medial degeneration (Table 5).

Synthesis of the histopathological data showed that patients with AA had milder degeneration of the media and, consequently, a lower frequency of dissection compared with the other group.

In both groups, the histopathological changes observed at the level of the ascending aorta from samples collected during surgical treatment were examined in association with the mortality rate.

**Figure 6:**
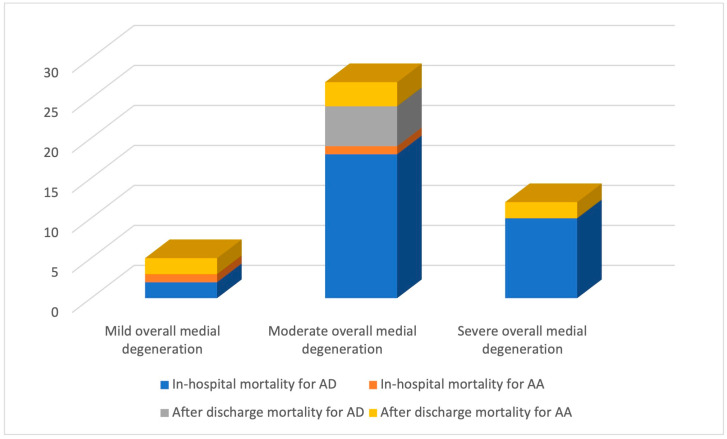
The link between histopathological score and mortality rate.

Regarding the relationship between predictor variables and the outcome variable, the analysis showed that for each unit increase in the histopathological score (i.e., an increase from mild to moderate or moderate to severe), the odds of in-hospital mortality increased by a factor of 3.073, and the odds of overall mortality increased by a factor of 2.9 (Table 6). A statistically significant link was found between the histopathological score, in-hospital mortality (*p* = 0.0029), and overall mortality (*p* = 0.0025; Figure 6).

## 3. Discussion

Aortic disease risks and predisposing variables are widely understood. This potentially life-threatening condition must be identified and managed promptly to prevent catastrophic consequences [13]. Our study identified clinical characteristics including age over 60 years, male gender, and comorbidities such as bicuspid aortic valve, pericardium effusion, or cardiac tamponade as more prevalent in the AD group than in the AA group. These risk factors might also influence the management of aortic diseases and can impact the mortality rates [1,6].

In patients with aortic disease, the mortality rate at various stages (pre-, peri-, and postoperative) remains high, and the time from diagnosis to treatment does not seem consistently brief [4,14,15]. Although postoperative complications could raise overall mortality, these factors are secondary to the primary cause, which is the rapid development of aortic disease. Complications following surgery did not prove to influence the mortality rate in our study. This data highlights the crucial role of adequate treatment after surgery and the importance of the histopathological score, evaluated in the aortic wall, in predicting evolution. The restant native thoraco-abdominal aorta segments remain vulnerable to the pre-existing risk factors that can influence the mortality rate.

Although it is widely accepted that the tissue of the aorta wall may undergo alterations, how these changes might influence patient outcomes is unclear. The importance of the consensus grading scale established by the Association for European Cardiovascular Pathology and the Society for Cardiovascular Pathology for daily practice is still poorly understood and needs more research [8,12,16,17].

Despite the unknown impact, it is universally agreed that histological changes influence patient outcomes [18,19]. Our study highlights the potential implications of these changes in mortality rates for patients who had recent surgical procedures for AD or AA.

The deep analysis performed in this study revealed significant differences between the two groups in MEMA-i modifications, with a moderate score for the AA group and a severe score for the AD group. AA samples with significant MEMA-t changes showed mild, moderate, and severe AD scores. Among cases with mild and severe scores for AA and moderate scores for AD, elastic fragmentation and loss of substantial changes were identified. AA with mild scores and AD with moderate or severe scores both influence smooth muscle cell nuclei loss. For minor scores in AA and moderate or severe scores in AD, laminar medial collapse alterations were observed.

Based on these findings, the AD and AA frequencies were positively correlated with the degree of medial degeneration identified under the microscope. AD is more likely to occur due to moderate and severe medial degeneration compared to cases of mild medial degeneration, which is more likely to result in AA. Consequently, medial degeneration is a major risk factor for both AA and AD [20].

Knowing the associated medical conditions of patients with aortic diseases allows us to determine their management before, during, and after surgery and decrease the mortality rate of these complex and life-threatening diseases. However, these conditions can also be correlated with histopathological changes, which are the basis of more detailed immunohistochemical and genetic studies in aortic pathology, including rheumatologic evaluation if the etiology is aortitis [21,22,23,24].

An important issue is the possible link between histological findings and the risk of mortality after an ascendant aortic replacement. Considering the patient’s age, gender, and pre-existing health conditions, as is done for preoperative anticoagulant therapy, might allow the patient’s postoperative management to be individualized to obtain a lower mortality rate [25,26,27,28,29].

To guide efforts to improve patient outcomes and decrease mortality rates, future research should be focused on these potential factors and detecting the fundamental mechanisms of their relationships with overall mortality [30].

The study also highlighted that both in-hospital and overall mortality significantly increased with each degree of histopathological score. This confirms the hypothesis that a relationship exists between histopathological changes and in-hospital and overall mortality rates, although the precise mechanisms leading to the association of the histopathology score with the probability of in-hospital mortality are not yet fully known [4].

The presence of aortic disease increases the risk of death in patients who reveal histopathological changes in intraoperatively harvested aortic tissue. This clinically relevant factor must be considered in the prevention program implemented for patients with this disease. Further studies should be conducted to accurately assess these results and analyze potential disagreements for better real-life application.

The degree to which certain risk factors and comorbidities are involved in aortic diseases is known, whereas others are poorly understood and require investigation [31,32,33,34,35]. Identifying those factors will be important for establishing screening and treatment programs that might prevent the disease’s progression and decrease the possible risk of AD [36].

### Limitations

The study was carried out in a single cardiac surgery department. Additionally, the follow-up time of six months after surgery was too short for a proper evaluation of the impact of histopathological changes on long-term survival.

Another limitation is that the reported mortality rate is quite high. The study was conducted in a tertiary center, where patients arrive from six distinct counties, and the time between diagnosis and surgical treatment was prolonged for many patients. Despite all surgical therapeutic efforts, these factors increased the revealed mortality rate.

## 4. Materials and Methods

Patients with AD hospitalized in the Emergency Unit were included in this study. In all consecutive cases, the ascending aorta segments obtained after surgical replacement were histopathologically examined and compared with those collected from patients who were admitted on an ongoing schedule and underwent surgical replacement for AA.

This prospective study included 108 consecutive patients diagnosed with aortic lesions (AD and AA), who received aortic replacement in a single tertiary cardiac surgery institution between 2019 and 2021. The follow-up time was six months.

Adult patients with AA or AD who submitted a signed informed consent for the surgical intervention and the publication of scientific data were included. The study was conducted following the Declaration of Helsinki and approved annually by the Ethical Committee of the George Emil Palade University of Medicine, Pharmacy, Sciences and Technology, Targu Mures, Romania (resolution 7225/07.10.2019, 878/23.04.2020, and 1359/10.05.2021).

Patients aged under 18 years and adult patients who refused to provide consent were excluded from the study. All data related to the patients were analyzed following ethical standards, and standard surgical operating protocols were applied.

In-hospital mortality refers to patients who died while receiving treatment during hospitalization. Overall mortality corresponds to the mortality rate of patients within the first six months of their follow-up period.

### 4.1. Histological Assessment

Standard protocols were used for processing the ascending aortic wall samples after 24 h of formalin fixation and then paraffin embedding. Each case was analyzed on HE and used three histologic stains: PAS Alcian, van Gieson–elastica, and trichrome.

Histological samples were assessed using the standards recommended by Halushka et al. [12]. Using as major criteria the presence of mucoid extracellular matrix accumulation (MEMA) with either intralamellar (MEMA-i) or translamellar (MEMA-t) extension, elastic fiber changes (fragmentation and loss), smooth muscle cell changes (nuclei loss and cell disarray), and collagen alteration (laminar medial collapse), based on their severity, the overall medial degeneration was scored as mild (score 1), moderate (score 2), or severe (score 3; Table 7). The histopathological samples were analyzed considering the type of aortic disease (AD or AA) and its microscopic aspects. The differences between the two groups were examined for in-hospital and overall mortality.

### 4.2. Statistical Tests and Standard Protocols

The statistical analysis included elements of descriptive statistics (frequency, percentage, mean, median, standard deviation) and elements of inferential statistics. The Shapiro-Wilk test was applied to determine the distribution of the analyzed data series. For median comparisons, the Mann Whitney test was applied. Chi-square and Fisher tests were applied to evaluate the relationships between qualitative variables. Furthermore, to quantify the relationship between predictor variables and the outcome variable, we performed a binary logistic regression for binary dependent variables. A *p*-value of 0.05 was selected as the level of significance. SPSS Statistics 28.0.0 software was used for the analysis.

## 5. Conclusions

Although the histopathological evaluation of the aortic wall is neglected in many centers, it can be a useful diagnostic tool and a predictor of both in-hospital and overall mortality, with a significant increase in mortality risk for every degree of histopathological score. Multicenter studies are necessary to evaluate the real impact of the histopathological assessment on the postoperative evolution of both AA and AD. Furthermore, developing national registers and rolling out prevention programs is necessary to decrease the mortality rate of patients with AD. Due to the surgical treatment involving a limited aortic segment, histopathological changes have significant clinical importance. Based on the changes revealed by the replaced aorta, a guideline might be elaborated for the follow-up of the patient and prevention of further AD.

## Figures and Tables

**Table 1:** Clinical characteristics of patients with aortic dissection and aortic aneurysm.

Parameter	Aortic Dissection (n = 52)	Aortic Aneurysm (n = 56)	OR	*p*-Value
Male	36 (69.23%)	42 (75%)	0.8654	0.5266
Female	16 (30.77%)	14 (25%)		
Age (mean ± SD) (median)	62.42 ± 12.79	57.20 ± 9.572	-	0.0072
Hospitalization days (mean ± SD)	8.769 ± 7.722	11.11 ± 11.20	-	0.1631

**Table 2:** The rate of complications in patients with aortic dissection and aortic aneurysm.

Parameter	Aortic Dissection (n = 52)	Aortic Aneurysm (n = 56)	OR	*p*-Value
**Preoperative**				
Pericardial effusion	21 (40.38%)	0 (0.00%)	77.127	<0.0001
Cardiac tamponade	10 (19.23%)	0 (0.00%)	27.918	0.0004
Malperfusion syndrome	2 (3.85%)	0 (0.00%)	5.594	0.2295
Pleural effusion	0 (0.00%)	1 (1.79%)	2.3186	0.9999
Acute kidney injury	6 (11.54%)	7 (12.50%)	0.9532	0.9999
Chronic kidney diseases	1 (1.92%)	2 (3.57%)	0.6863	0.9999
Stroke	1 (1.92%)	0 (0.00%)	2.098	0.4815
Syncope	2 (3.85%)	0 (0.00%)	5.594	0.2295
Systemic hypertension	32 (61.54%)	18 (32.14%)	3.378	0.0036
Bicuspid aortic valve	17 (30.36%)	3 (5.77%)	7.120	0.0011
Aortic valve regurgitation	32 (61.54%)	27 (48.21%)	1.1719	0.1809
Aortic valve stenosis	2 (3.85%)	7 (12.50%)	0.2800	0.1638
Atrial fibrillation	28 (50.00%)	7 (13.46%)	6.429	<0.0001
**Postoperative**				
Days in the ICU (mean ± SD)	2.750 ± 2.317	4.268 ± 5.786	-	0.6173
Intubation time-hours (mean ± SD)	36.63 ± 51.31	65.90 ± 130.4	-	0.2969
Hepatic dysfunction	18 (34.62%)	23 (41.07%)	0.7596	0.5541
Renal dysfunction	11 (21.15%)	15 (26.79%)	0.7333	0.5100
Hemofiltration	7 (13.46%)	11 (19.64%)	0.6364	0.4461
Systemic infection	2 (3.85%)	4 (7.14%)	0.5200	0.6799
Reintervention	10 (19.23%)	7 (12.50%)	1.667	0.4304

**Table 3:** Microscopic scores in aortic dissection vs. aortic aneurysm samples.

Histological Medial Degenerative Change	Degenerative Degree	Aortic Dissection (n = 52)	Aortic Aneurysm (n = 56)	*p*-Value
Interlamellar (MEMA-i)	Mild	0 (0.00%)	6 (10.71%)	<0.0001
	Moderate	9 (17.31%)	24 (42.86%)	
	Severe	43 (82.69%)	26 (46.43%)	
Translamellar (MEMA-t)	Mild	4 (7.69%)	20 (40.00%)	0.0001
	Moderate	40 (79.62%)	24 (48.00%)	
	Severe	8 (15.38%)	6 (12.00%)	
Elastic fragmentation or loss	Mild	11 (21.15%)	29 (54.72%)	0.0011
	Moderate	37 (71.15%)	20 (37.74%)	
	Severe	4 (7.69%)	4 (7.55%)	
Smooth muscle cell nuclei loss	Mild	26 (50.98%)	26 (70.27%)	<0.0001
	Moderate	23 (45.10%)	10 (27.03%)	
	Severe	2 (3.92%)	1 (2.70%)	
Laminar medial collapse	Mild	22 (45.83%)	24 (60.00%)	0.0223
	Moderate	22 (45.83%)	14 (35.00%)	
	Severe	4 (8.33%)	2 (5.00%)	

**Table 4:** Subdivision of the microscopic score in aortic dissection vs. aortic aneurysm.

Histological Medial Degenerative Change	Degenerative Degree	Subdivision	Aortic Dissection (n = 52)	Aortic Aneurysm (n = 56)	*p*-Value
Interlamellar (MEMA-i)	Mild	Multifocal	0 (0.00%)	4 (100%)	0.2378
		Focal	0 (0.00%)	2 (100%)	
	Moderate	Multifocal	9 (30.00%)	21 (70.00%)	0.5447
		Focal	0 (0.00%)	3 (100%)	
	Severe	Multifocal	40 (60.61%)	26 (39.39%)	0.2852
		Focal	3 (100%)	0 (0.00%)	
Translamellar (MEMA-t)	Mild	Multifocal	3 (23.08%)	10 (76.92%)	0.5963
		Focal	1 (9.09%)	10 (90.91%)	
	Moderate	Multifocal	29 (69.05%)	13 (30.95%)	0.1768
		Focal	11 (50.00%)	11 (50.00%)	
	Severe	Multifocal	4 (44.44%)	5 (55.56%)	0.3007
		Focal	4 (80.00%)	1 (20.00%)	
Elastic fragmentation or loss	Mild	Multifocal	5 (31.32%)	11 (68.57%)	0.7275
		Focal	6 (25.00%)	18 (75.00%)	
	Moderate	Multifocal	22 (61.11%)	14 (38.89%)	0.5675
		Focal	15 (71.43%)	6 (28.57%)	
	Severe	Multifocal	1 (20.00%)	4 (80.00%)	0.1429
		Focal	3 (100%)	0 (0.00%)	
Smooth muscle cell nuclei loss	Mild	Multifocal	12 (50.00%)	12 (50.00%)	0.9999
		Focal	14 (50.00%)	14 (50.00%)	
	Moderate	Multifocal	12 (63.16%)	7 (36.84%)	0.4551
		Focal	11 (78.57%)	3 (21.43%)	
	Severe	Multifocal	2 (100%)	0 (0.00%)	-
		Focal	0 (0.00%)	1 (100%)	
Laminar medial collapse	Mild	Multifocal	14 (73.68%)	5 (26.32%)	0.0063
		Focal	8(29.63%)	19(70.37%)	
	Moderate	Multifocal	13 (61.90%)	8 (38.10%)	0.9999
		Focal	9 (60.00%)	6 (40.00%)	
	Severe	Multifocal	2 (100%)	0 (0.00%)	0.4667
		Focal	2 (50.00%)	2 (50.00%)	

**Table 5:** Overall medial degeneration in aortic dissection vs. aortic aneurysm.

Severity	Aortic Dissection	Aortic Aneurysm	*p*-Value
Mild (n = 26)	2 (7.69%)	24 (92.31%)	<0.0001
Moderate (n = 64)	39 (60.94%)	25 (39.06%)	
Severe (n = 18)	11 (61.11%)	7 (38.89%)	

**Table 6:** Analysis of mortality in relation to histopathological score.

In-Hospital Mortality					
Predictors	B	Exp(B)	95% CI for EXP(B)		*p*-value
			Lower	Upper	
Histopathologic score	1.123	3.073	1.467	6.439	0.0029
		**Overall Mortality**			
Predictors	B	Exp(B)	95% CI for EXP(B)		*p*-value
			Lower	Upper	
Histopathologic score	1.065	2.900	1.455	5.779	0.0025

**Table 7:** Assessment criteria used for the scoring of aortic medial degeneration.

Mucoid Extracellular Matrix Accumulation, Intralamellar (MEMA-i)		Mucoid Extracellular Matrix Accumulation, Translamellar (MEMA-t)		Elastic Fiber Fragmentation and Loss		Loss of Smooth Muscle Cell Nuclei		Laminar Medial Collapse		Overall Medial Degeneration
Absent		Absent		Absent		Absent		Absent		ABSENT (score 0)
Mild		Mild		Mild		Mild		Mild		MILD (score 1)
Focal	Multifocal	Focal	Multifocal	Focal	Multifocal	Focal	Multifocal	Focal	Multifocal	
Moderate		Moderate		Moderate		Moderate		Moderate		MODERATE (score 2)
Focal	Multifocal	Focal	Multifocal	Focal	Multifocal	Focal	Multifocal	Focal	Multifocal	
Severe		Severe		Severe		Severe		Severe		SEVERE (score 3)
Focal	Multifocal	Focal	Multifocal	Focal	Multifocal	Focal	Multifocal	Focal	Multifocal	

## Data Availability

The data supporting this study’s findings are available from the corresponding author upon request.
